# Dentofacial effects of miniscrew-anchored maxillary protraction on prepubertal children with maxillary deficiency: a randomized controlled trial

**DOI:** 10.1186/s40510-023-00473-4

**Published:** 2023-06-12

**Authors:** Ahmed Mohamed Kamel, Nour Eldin Tarraf, Ahmed Maher Fouda, Ahmad Mohammed Hafez, Ahmed El-Bialy, Benedict Wilmes

**Affiliations:** 1 Department of Orthodontics, Faculty of Dentistry, Mansoura University, 1 El-Gomhouria St, Mansoura, Dakahlia Governorate 35516 Egypt; 2 Department of Orthodontics, Faculty of Medicine and Health, Discipline of Orthodontics and Paediatric Dentistry, Sydney Dental School, University of Sydney, Sydney, Australia; 3 Department of Orthodontics, University of Düsseldorf, Düsseldorf, Germany

**Keywords:** Skeletal anchorage, Hybrid hyrax, Class III malocclusion, Maxillary protraction

## Abstract

**Background:**

The introduction of bone-anchored maxillary protraction eliminated the side effects of facemask in the early treatment of patients with maxillary retrusion. This study aimed to evaluate the effects of miniscrew-anchored maxillary protraction (MAMP) and compare them with the growth changes in an untreated control group in growing patients with Class III malocclusion.

**Methods:**

Forty growing patients with Class III malocclusion and retrognathic maxilla were randomly allocated into two groups: treated and control groups. In the treated group, patients were treated with full-time intermaxillary Class III elastics (C3E) anchored by a hybrid hyrax (HH) in the maxilla and a bone-supported bar in the mandible. Protraction was stopped after obtaining a positive overjet. Cephalometric radiographs were acquired before and after the treatment. Data were statistically analyzed on an intention-to-treat basis. Intergroup comparisons were also made using analysis of covariance with the readings at T0 as a covariate.

**Results:**

Forty patients agreed to participate, and 30 of them completed the study (treated group, *n* = 17; control group, *n* = 13). The average treatment duration was 11.9 months. MAMP resulted in a significant maxillary advancement (A-VR, 4.34 mm) with significant control over the mandibular growth. No significant increase in the mandibular plane angle was found in the treated group compared with the control group. The upper and lower incisors showed significant protrusion in the treated group.

**Conclusions:**

Within the limitations of this study and high attrition rate, the MAMP protocol can effectively increase maxillary forward growth with good control over the growth of the mandible antero-posteriorly and vertically.

## Background

Skeletal Class III malocclusion is considered one of the most challenging problems to manage in orthodontics with early intervention usually required. Sixty-seven percent of patients with Class III malocclusion present with maxillary retrognathism [1].

Facemask (FM) is the most common modality used for maxillary protraction in the early mixed dentition [2]. The indirect anchorage of the FM to the maxilla through the dentition has many disadvantages, including mesialization of the maxillary dentition with incisor proclination and mandibular incisor retroclination [3, 4]. The FM treatment is also accompanied by rotations of the maxilla and mandible that increases the lower anterior facial height. Moreover, the sagittal maxillary changes tend to be insignificant at 3-year follow-up [5].

De Clerck et al. [6] replaced the extraoral traction forces of the FM by intraoral Class III elastics (C3E) attached to infrazygomatic miniplates and symphyseal miniplates in the mandible. The technique was described as bone-anchored maxillary protraction (BAMP). Wilmes et al. used C3E between a hybrid hyrax (HH) in the maxilla and mentoplates in the mandible [7–9]. Several studies have presented a modified BAMP technique by replacing the anchor plates with orthodontic mini-implants (OMI), which was known as miniscrew-anchored maxillary protraction (MAMP) [10–14].

The alternate rapid maxillary expansion and constriction (Alt-RAMEC) protocol was used to increase the orthopedic effect of the protraction appliances by loosening the circummaxillary sutures [15, 16].

To our knowledge, the literature lacks randomized controlled studies that evaluate the effects of the MAMP in growing Class III patients compared to changes with growth in an untreated control group. This study aimed to evaluate the skeletal, dentoalveolar, and soft tissue effects of MAMP and compare them with the growth changes in the untreated control group.

## Methods

### Trial design

This was a parallel-group randomized controlled trial with a 1:1 allocation ratio.

### Participants, eligibility criteria, and settings

Forty growing patients with Class III malocclusion were recruited for this study from the clinic of the Orthodontic Department, Faculty of Dentistry, Mansoura University, Egypt. This study was approved by the dental research ethics committee (code no. A16260219). Patients were enrolled based on the following criteria: (1) skeletal Class III (ANB < 0, Wits < − 2) with maxillary retrusion (A-N Perp < 0), (2) growing patients according to the cervical vertebral maturation method (CS1–CS3), (3) late mixed or early permanent dentition with an anterior crossbite, (4) erupted mandibular canines. Exclusion criteria included patients with syndromes, craniofacial anomalies, or previous orthodontic treatment.

### Interventions

The research protocol was explained to the 40 candidates who met the inclusion criteria. Written consent forms to participate in the study were signed by the parents. Patients were randomly assigned to two groups. In group 1 (*n* = 20), C3E was used attached to a HH expander in the maxilla to a bone-anchored mandibular bar. In group 2 (*n* = 20, control group), no intervention was provided to rule out growth changes.

**Fig. 1:**
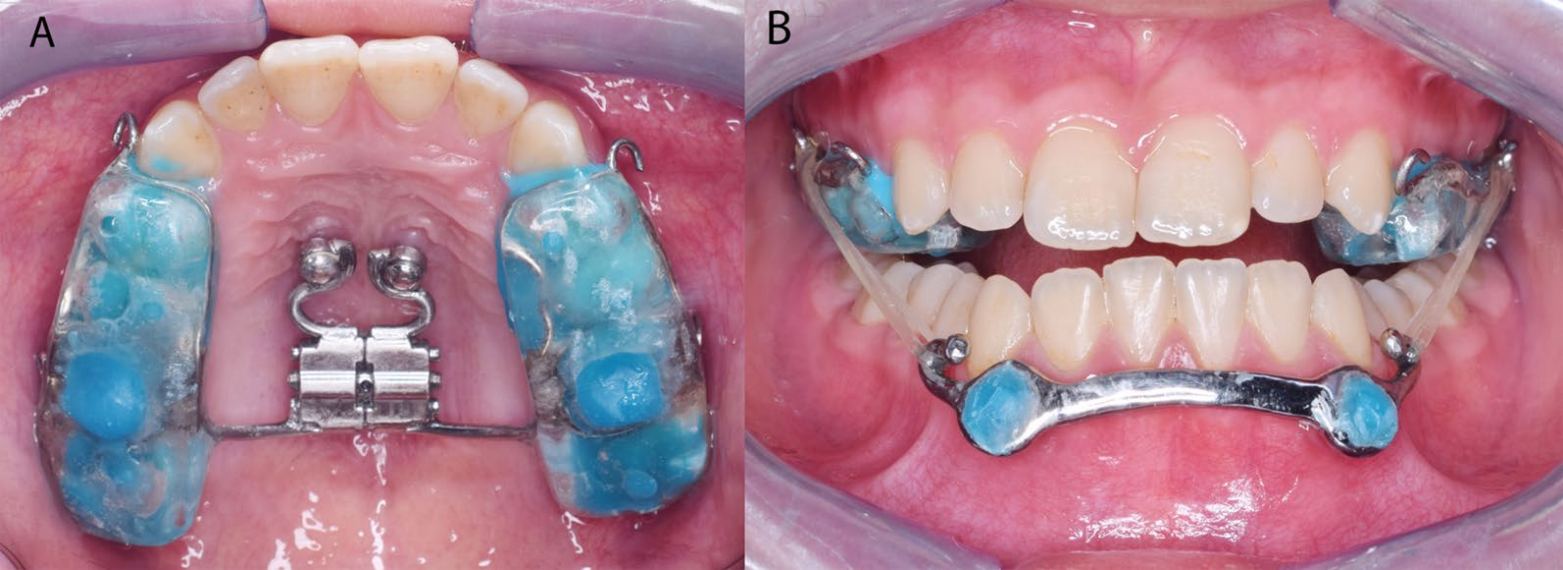
**A** Hybrid hyrax appliance in situ. **B** Class III elastics were attached from the posterior hooks in the maxilla to those of the mandibular bar

In the maxilla, two OMIs (8 mm length, 1.8 mm diameter; 3 M ESPE Dental Products, St. Paul, MN, USA) were placed 2–3 mm lateral to the median palatine suture, distal to the third palatine rugae, and at approximately 20–30° distal angulation [17]. HH was constructed using an expansion screw (Hyrax^®^; Dentaurum, Ispringen, Germany) with the anterior arms welded to the OMI caps and the posterior arms to the molar bands. Hooks for the C3E were welded on the buccal surface of the molar bands. The HH was fabricated with posterior bite blocks to eliminate any occlusal interference. The HH was assessed intraorally for the complete passive seating of the appliance and then cemented (Fig. 1).

In the mandible, two OMIs (8 mm length, 1.5 mm diameter, 2 mm transmucosal; Morelli, S.B, Brazil) were placed at the mucogingival line between the mandibular canine and lateral incisor at 20–30° apical to the occlusal plane. A custom-made bar was fabricated with two hooks and two rings to fit precisely over the OMI heads. The bar was checked intraorally for the complete passive seating of the rings on the OMI without tissue impingement. After cementation, it was evaluated for stability under the forces of the elastics.

**Fig. 2:**
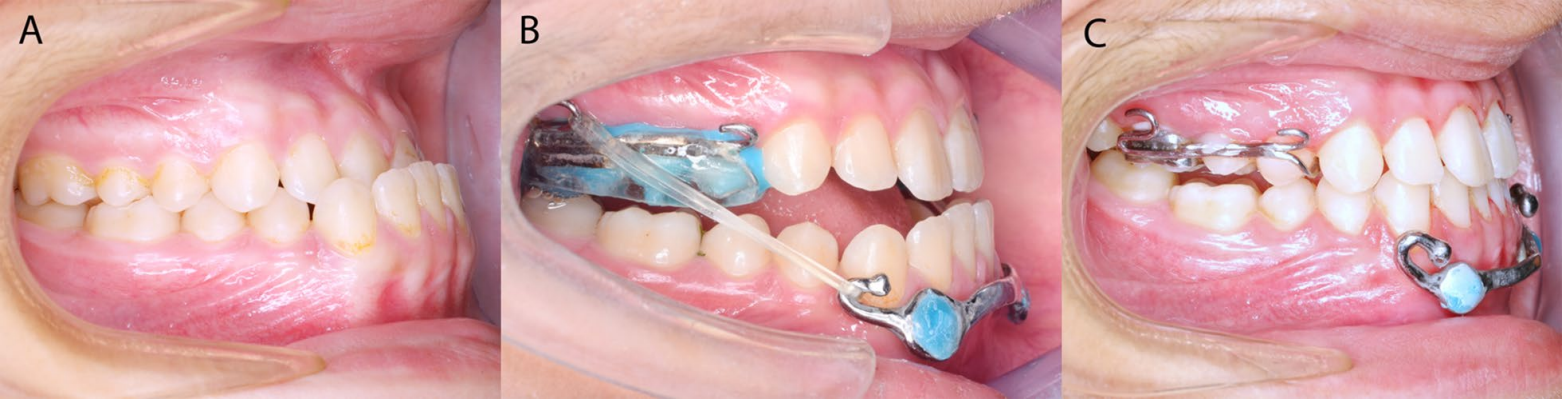
**A** Before, **B** with Class III elastics, and **c** after maxillary protraction by the MAMP protocol

In the Alt-RAMEC phase, the expansion screw was activated two-quarter turns twice a day for 7 days. Afterward, the screw was reversed with the same frequency for 7 days. This process of alternate expansion and contraction was conducted for 9 weeks ending with a week of expansion. Intermaxillary C3E were attached on each side from the posterior hooks in the HH to those of the bar initiating the maxillary protraction phase (Fig. 1). The forces were adjusted to 100 g on each side as initial force and increased to 200 g in the second month. The instructions entailed full-time wear of the elastics and replacement every 12 h. The bite blocks were removed immediately after the overjet correction. The protraction phase was terminated when the patients reached a 2 to 3 mm positive overjet (Fig. 2).

### Outcomes

**Fig. 3:**
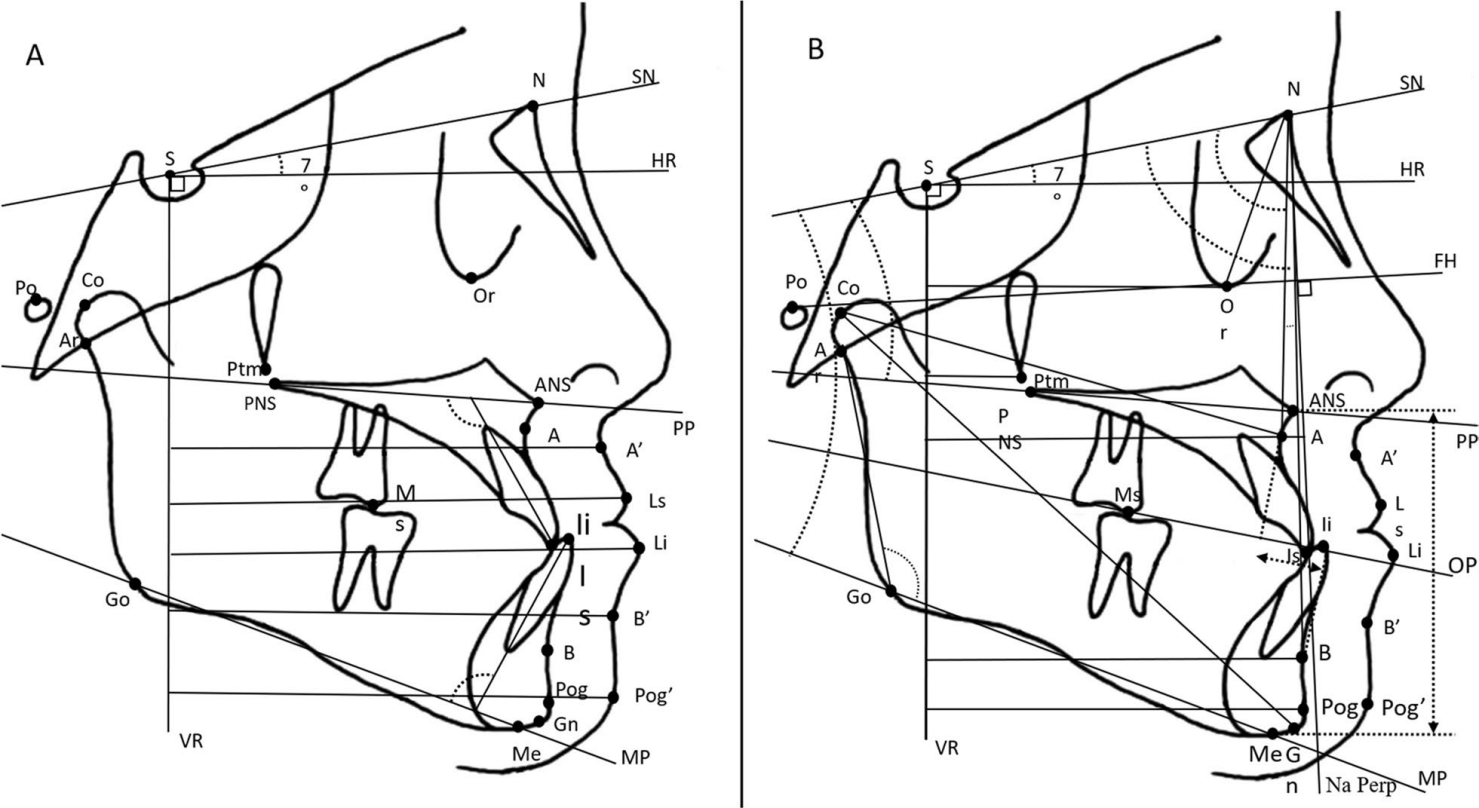
**A** Skeletal measurements. **B** Soft tissue and dentoalveolar measurements

**Fig. 4:**
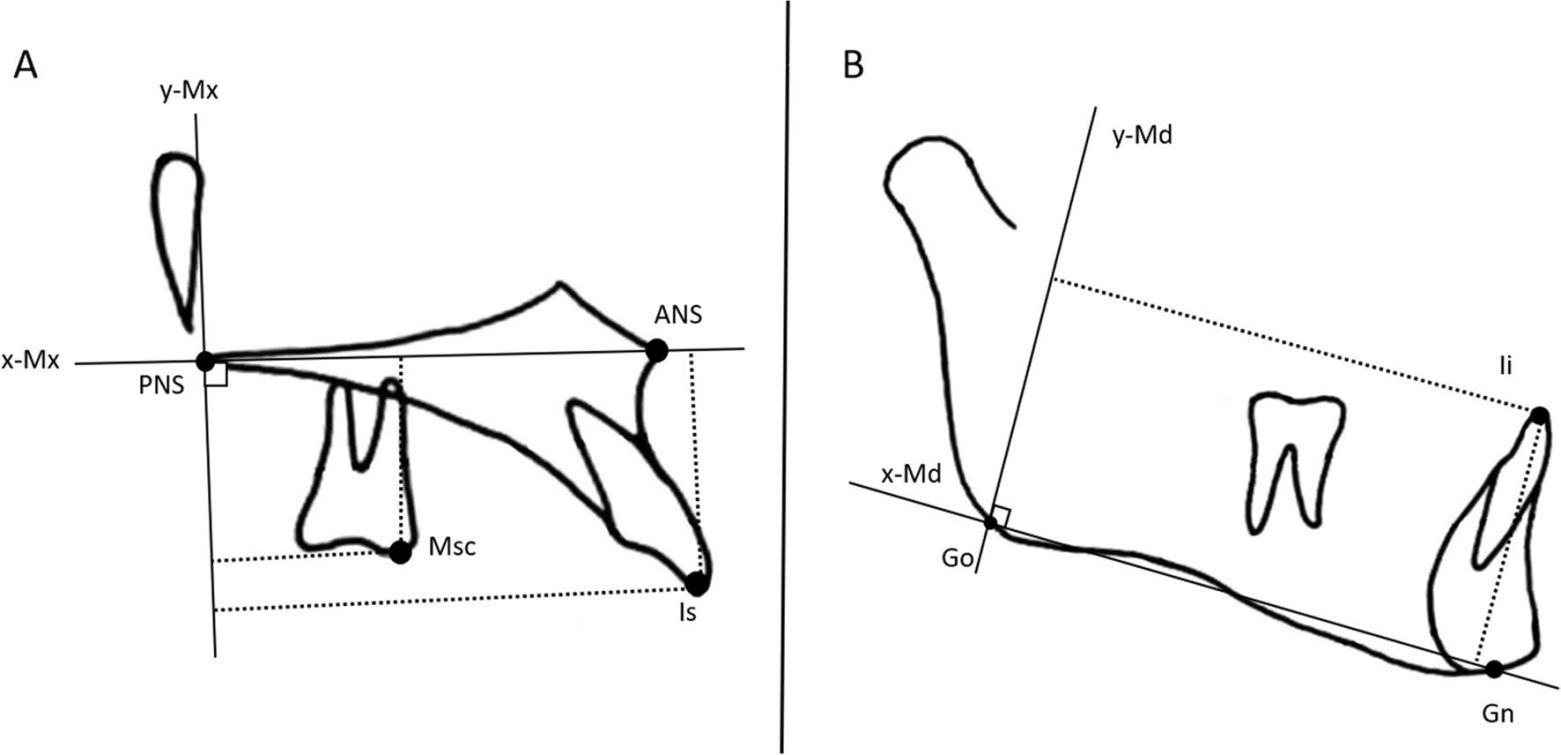
**A** Local maxillary superimposition and **B** local mandibular superimposition with dental measurements

The primary outcome was the forward advancement of the maxilla after the follow-up period detected by the change in the position of the A-point. The secondary outcomes were other cephalometric changes. Skeletal, dental, and soft tissue variables were measured before (T0) and after (T1) the protraction or observation periods using lateral cephalometric radiographs (MAMP group, 11.9 ± 2.1 months; Control group = 12 months). All landmarks and measurements were adopted from previous studies (Figs. 3 and 4, Tables 1 and 2) [18–20]. Cephalometric skeletal, dentoalveolar, and soft tissue variables were measured using AudaxCeph software (version 3.4; Ljubljana, Slovenia).

**Table 1:** Definitions of cephalometric reference points, lines, and planes

Name	Symbol	Definition
Sella	S	The center of the hypophysial fossa (sella turcica)
Nasion	N	The most anterior point of the fronto-nasal suture
Orbitale	Or	Lowest point on the inferior margin of the orbit
Porion	Po	Uppermost point of bony external auditory canal
Anterior nasal spine	ANS	The anterior tip of the sharp bony process of the maxilla in the midsagittal plane
Posterior nasal spine	PNS	The most posterior aspect of the palatine bone in the midsagittal plane
Subspinale	A	The deepest point in the midline concavity of the anterior maxilla between the ANS and the alveolar crest (prosthion)
Supramentale	B	The deepest point in the midline concavity of the anterior mandible between the alveolar crest (infradentale) and pogonion
Pogonion	Pg	The most prominent point on the chin
Gnathion	Gn	The most anterior inferior point of the bony chin
Menton	Me	The most inferior point on the mandibular symphysis
Gonion	Go	Most posterior inferior point on angle of mandible, located by bisecting the angle formed by the ramal and mandibular planes
Articulare	Ar	Intersection of posterior border of ramus and inferior border of occipital bone
Condylion	Co	Most posterior superior point on mandibular condyle
Pterygomaxillary fissure	Ptm	The intersection of the anterior and posterior walls of the pterygomaxillary fissure inferiorly
Maxillary incisor tip	Is	The incisal tip point of the most prominent maxillary central incisor
Mandibular incisor tip	Ii	The incisal tip point of the most prominent mandibular central incisor
Infradentale	Id	The most anterosuperior point on the labial crest of the mandibular alveolar process
Molar superius mesial cusp	Ms	The mesio-buccal cusp tip of the maxillary first permanent molar
Soft tissue subspinale	A’	The point of greatest concavity in the midline of the upper lip between subnasale and labrale superius
Labrale superius	Ls	The most anterior point on the convexity of the upper lip
Labrale inferius	Li	The most anterior point on the convexity of the lower lip
Soft tissue submentale	B’	The point of greatest concavity in the midline of the lip between labrale inferius and soft tissue pogonion
Soft tissue pogonion	Pg’	The most prominent or anterior point on the soft tissue chin in the midsagittal plane
Horizontal reference plane	HR	A line established by rotating 7° clockwise from sella-nasion plane
Vertical reference plane	VR	A vertical line passing through sella and perpendicular to the HR plane
Sella-Nasion line	SN	Reference line joining sella and nasion points
Frankfort Horizontal	FH	Reference line joining porion and orbitale points
Nasion perpendicular	Na Perp	Nasion perpendicular line to FH plane
Occlusal plane	OP	Plane drawn through the region of overlapping cusps of first premolars and first molars
Palatal plane (Maxillary horizontal reference line)	PP (*x* − Mx)	Reference line joining anterior nasal spine and posterior nasal spine
Mandibular plane	MP	Reference line joining menton and gonion
Mandibular horizontal reference line	*x − *Md	Reference line joining gnathion and gonion
Maxillary vertical reference line	*y* − Mx	A vertical line passing through the PNS and perpendicular to *x* − Mx
Mandibular vertical reference line	*y* − Md	A vertical line passing through the gonion, perpendicular to the *x* − Md

**Table 2:** Cephalometric skeletal, dental, and soft tissue measurements

Variable	Definition
Skeletal measurements	
Sagittal measurements	
Linear measurements (mm)	
Or-VR	Perpendicular distance from Orbitale to VR
Ptm-VR	Perpendicular distance from Ptm to VR
A-VR	Perpendicular distance from A to VR
B-VR	Perpendicular distance from B to VR
Pg-VR	Perpendicular distance from Pogonion to VR
A-Na Perp	Perpendicular distance from A-point to Nasion Perpendicular line to FH plane
Wits appraisal	The distance between AO and BO projections of points A and B to Occlusal Plane
Co-A	Maxillary length; the distance between Condylion and point A
Co-Gn	Mandibular length; the distance between Condylion and Gnathion
Angular measurements (°)	
SNA	Angle between the anterior cranial base and (Nasion-point A) line
SNB	Angle between the anterior cranial base and (Nasion-point B) line
ANB	Angle between (Nasion-point A) and (Nasion-point B) lines, SNA minus SNB
SNO	Angle between the anterior cranial base and (Nasion-Orbitale) line
Vertical measurements	
Linear measurements (mm)	
ANS-Me	Lower facial height (LFH)
Angular measurements (°)	
SN-MP	Mandibular plane angle relative to the SN line
SN-PP	Maxillary plane angle relative to SN line
Ar-Go-Me	Gonial angle between (Articulare-Gonion) line and (Gonion-Menton) line
Dental measurements	
Sagittal measurements	
Linear measurements (mm)	
Is-yMx	Perpendicular distance from maxillary incisor tip to yMx
Ms-yMx	Perpendicular distance from mesio-buccal cusp tip of the maxillary first permanent molar to yMx
Ii-yMd	Perpendicular distance from mandibular incisor tip to yMd
Overjet	Horizontal overlap of the maxillary central incisors over the mandibular central incisors
Angular measurements (°)	
Is-PP	Angle between the long axis of the maxillary incisor and maxillary plane
Ii-MP	Angle between the long axis of the mandibular incisor and the mandibular plane
Vertical measurements	
Linear measurements (mm)	
Is-xMx	Perpendicular distance from maxillary incisor tip to xMx
Ms-xMx	Perpendicular distance from mesio-buccal cusp tip of the maxillary first permanent molar to xMx
Ii-xMd	Perpendicular distance from mandibular incisor tip to xMd
Overbite	Vertical overlap between the maxillary central incisors and the mandibular central incisors
Soft tissue linear measurements (mm)	
A’-VR	Perpendicular distance from A’ to VR
Ls-VR	Perpendicular distance from labrale superius to VR
Li-VR	Perpendicular distance from labrale inferius to VR
B’-VR	Perpendicular distance from B’ to VR
Pg’-VR	Perpendicular distance from soft tissue Pogonion to VR

### Sample size calculation

The sample size was estimated by G*Power software (version 3.1.9.4; Kiel University, Germany) according to previous studies [19, 20] (90% power, 5% significance level, two-tailed test). The A-point was advanced by 2.67 ± 1.49 mm and 1.18 ± 0.6 mm in the treated and control groups, respectively. The calculated sample size was 14 in each group and increased to 20 patients to address possible dropouts.

### Randomization and allocation concealment

Randomization was completed using stratified permuted block randomization according to sex. The participants were divided into two strata: male and female. Blocked randomization was used in each stratum. The randomization sequence was generated using PASS 2021 software (version 21.0.2; NCSS, Kaysville, UT, USA). The randomization list was kept with a person who was not included in the trial.

### Blinding

Blinding of either clinician or patients was not applicable during the trial. However, the statistician was blinded during data analysis.

### Statistical analysis

Statistical analyses were conducted using SPSS software (version 26.0; IBM Corp., Armonk, NY, USA). Data were tested for normality using Shapiro–Wilk’s test (*p* > 0.05) and z-scores of skewness and kurtosis (within ± 2.58). The presence of significant outliers was tested by inspecting boxplots. Independent-samples t test was used to compare the groups in regard to the treatment changes. Intention-to-treat analysis using multiple imputation was performed to deal with the missing data of the dropouts. Five datasets were generated using the SPSS software. By applying Rubin’s rule, the analysis was performed based on the pooled results of the 5 datasets. Intergroup comparisons were also made using the analysis of covariance with the readings at T0 as a covariate (intention-to-treat, per-protocol). The alpha level for all statistical tests was set at *p* < 0.05.

### Error of the method

Fifteen randomly selected cephalometric radiographs were carefully checked twice, 2 weeks apart, by one investigator to evaluate intraobserver errors. The intraclass correlation coefficient values ranged from 0.89 to 0.97 for all variables. These values indicate that the measurements were reliable.

## Results

### Participant flow

**Fig. 5:**
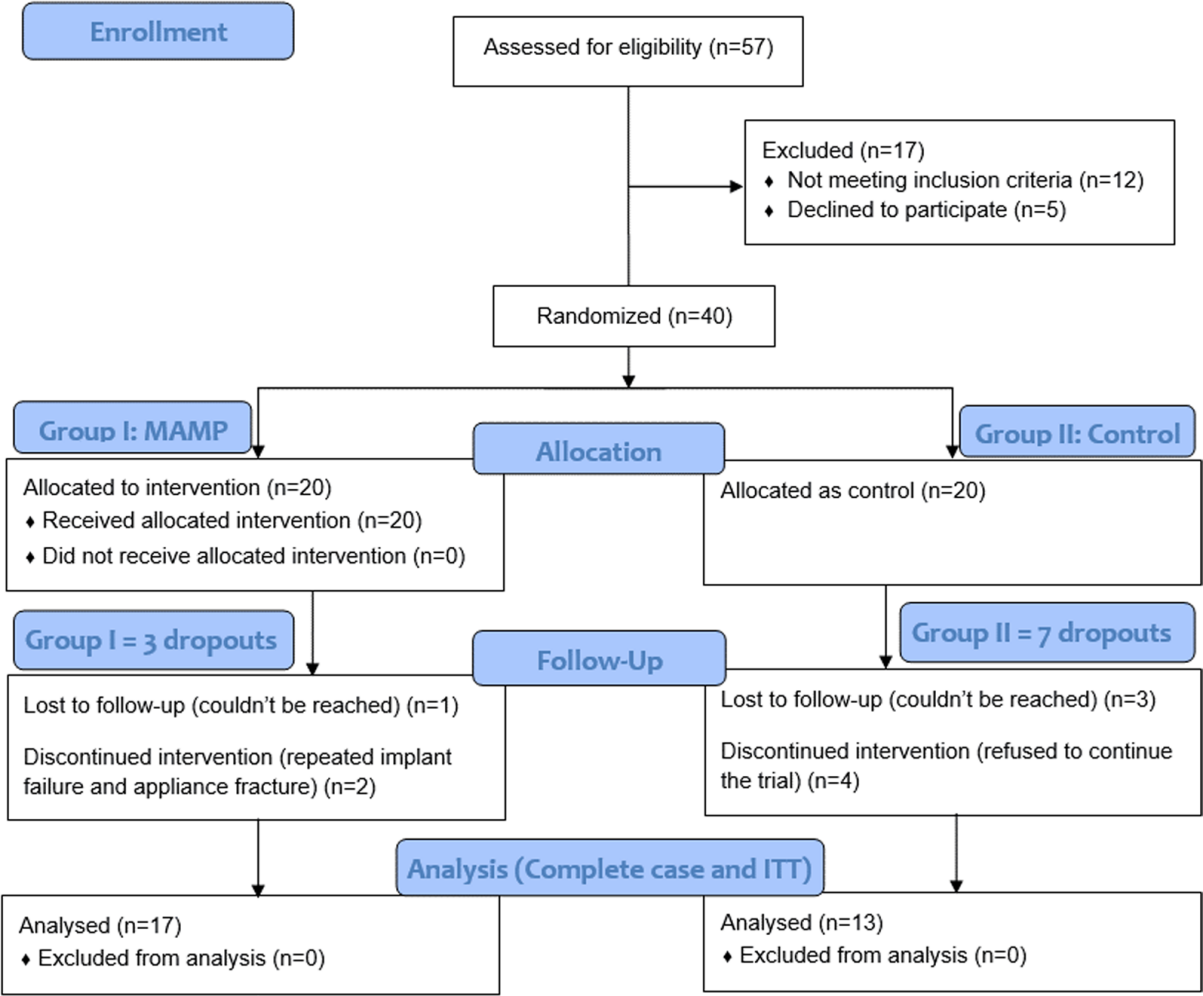
CONSORT diagram illustrating the participants flow in the study

The CONSORT diagram demonstrates the flow of the patients through the trial (Fig. 5).

### Baseline data

Table 3 shows the baseline characteristics and follow-up durations of the groups. Cephalometric variables in each group at T0 are listed in Table 4.

**Table 3:** Baseline data of the two groups

Characteristic	Treated group	Control group
*Sex* *N* (%)		
Male	9 (52.9%)	8 (61.5%)
Female	8 (47.1%)	5 (38.5%)
*Age (years)*		
Mean ± SD	11.3 ± 1.1	11.5 ± 1.2
*Duration (months)*		
Mean ± SD	11.9 ± 2.1	12

**Table 4:** Pre-treatment mean values of all parameters in each group at T0

Parameters	Treated group Mean ± SD	Control group Mean ± SD
Or-VR (mm)	51.11 ± 2.09	50.40 ± 1.94
Ptm-VR (mm)	17.77 ± 1.39	18.12 ± 1.52
A-VR (mm)	56.33 ± 3.32	56.11 ± 4.01
B-VR (mm)	57.52 ± 2.64	56.36 ± 3.13
Pg-VR (mm)	58.15 ± 2.02	58.19 ± 2.75
A-NPerp (mm)	− 3.35 ± 0.58	− 3.41 ± 0.61
Wits (mm)	− 5.05 ± 0.96	− 4.77 ± 0.85
Co-A (mm)	74.38 ± 2.29	73.96 ± 2.37
Co-Gn (mm)	102.74 ± 2.55	102.45 ± 2.24
SNA (°)	77.38 ± 1.09	77.48 ± 0.79
SNB (°)	80.87 ± 1.01	80.68 ± 0.89
ANB (°)	− 3.48 ± 1.50	− 3.20 ± 1.22
SNO (°)	54.97 ± 2.02	54.72 ± 1.62
ANS-Me (mm)	55.31 ± 3.22	54.03 ± 3.12
SN-MP (°)	35.26 ± 2.87	34.53 ± 2.40
SN-PP (°)	11.48 ± 1.69	10.80 ± 1.21
Ar-Go-Me (°)	132.42 ± 2.21	132.34 ± 2.40
Is-yMx (mm)	47.18 ± 2.66	46.79 ± 2.29
Ms-yMx (mm)	18.74 ± 2.13	18.40 ± 2.16
Ii-yMd (mm)	58.98 ± 2.47	58.08 ± 2.32
Overjet (mm)	− 3.48 ± 0.77	− 3.56 ± 0.61
Is-PP (°)	116.32 ± 3.21	115.56 ± 2.86
Ii-MP (°)	86 ± 3.56	86.85 ± 3.87
Is-xMx (mm)	25.58 ± 2.38	24.47 ± 2.79
Ms-xMx (mm)	18.79 ± 1.67	18.26 ± 1.38
Ii-xMd (mm)	36.21 ± 2.63	36.22 ± 2.71
Overbite (mm)	3.28 ± 1.15	3.20 ± 1.24
A’-VR (mm)	73.10 ± 2.12	72.79 ± 2.80
Ls-VR (mm)	78.70 ± 2.62	78.15 ± 2.64
Li-VR (mm)	78.69 ± 2.88	78.44 ± 3.04
B’-VR (mm)	71.22 ± 3.19	70.76 ± 3.33
Pg’-VR (mm)	74.06 ± 4.13	73.10 ± 4.32

### Numbers analyzed for each outcome

Table 5 shows a comparison of the mean cephalometric changes among the groups based on a complete case analysis. Table 6 demonstrates intergroup comparisons of adjusted means for post-treatment cephalometric measurements with pre-treatment measurements as covariates on an intention-to-treat basis. Comparisons based on complete case analysis are presented in Table 7.

**Table 5:** Comparison of cephalometric, skeletal, dental, and soft tissue mean changes among the groups

Parameter	Treated group Mean (SD)	Control group Mean (SD)	MD	95% CI	*P* value
Or-VR (mm)	3.37 ± 0.39	0.73 ± 0.32	2.63	2.35–2.90	< 0.001*
Ptm-VR (mm)	2.51 ± 0.247	0.56 ± 0.16	1.95	1.78–2.11	< 0.001*
A-VR (mm)	4.34 ± 0.51	0.87 ± 0.34	3.46	3.12–3.80	< 0.001*
B-VR (mm)	− 0.26 ± 0.41	1.37 ± 0.47	− 1.64	− 1.98 to − 1.31	< 0.001*
Pg-VR (mm)	− 0.30 ± 0.43	1.50 ± 0.41	− 1.80	− 2.13 to − 1.48	< 0.001*
A-NPerp (mm)	5.10 ± 0.49	0.35 ± 0.42	4.75	4.39–5.10	< 0.001*
Wits (mm)	5.27 ± 1.07	0.28 ± 0.45	4.99	4.34–5.64	< 0.001*
Co-A (mm)	4.54 ± 0.65	1.10 ± 0.39	3.44	3.02–3.87	< 0.001*
Co-Gn (mm)	1.47 ± 0.25	2.12 ± 1.12	− 0.65	− 1.22 to − 0.07	0.063
SNA (°)	4.64 ± 0.95	0.42 ± 0.21	4.21	3.66–4.77	< 0.001*
SNB (°)	− 0.25 ± 0.47	1.03 ± 0.59	− 1.29	− 1.70 to − 0.89	< 0.001*
ANB (°)	4.90 ± 1.31	− 0.61 ± 0.55	5.51	4.72–6.31	< 0.001*
SNO (°)	2.79 ± 0.50	0.30 ± 0.18	2.49	2.18–2.79	< 0.001*
ANS-Me (mm)	1.26 ± 0.40	0.76 ± 0.41	0.50	0.20–0.81	0.002*
SN-MP (°)	0.86 ± 1.07	0.49 ± 0.58	0.37	− 0.31 to 1.04	0.242
SN-PP (°)	0.07 ± 0.99	− 0.27 ± 0.83	0.35	− 0.36–1.10	0.320
Ar-Go-Me (°)	− 2.32 ± 0.81	− 0.13 ± 0.52	− 2.19	− 2.71 to − 1.65	< 0.001*
Is-yMx (mm)	2.38 ± 0.59	0.85 ± 0.29	1.53	1.19–1.87	< 0.001*
Ms-yMx (mm)	1.03 ± 0.44	0.50 ± 0.23	0.53	0.25–0.81	< 0.001*
Ii-yMd (mm)	2.10 ± 0.38	0.47 ± 0.17	1.63	1.41–1.85	< 0.001*
Overjet (mm)	5.97 ± 0.65	0.50 ± 0.14	5.47	5.12–5.81	< 0.001*
Is-PP (°)	2.31 ± 2.57	0.36 ± 0.20	1.95	0.48–3.42	0.011*
Ii-MP (°)	1.19 ± 0.59	0.58 ± 0.35	0.61	0.23–0.99	0.003*
Is-xMx (mm)	0.61 ± 0.25	0.45 ± 0.22	0.15	− 0.03 to 0.33	0.095
Ms-xMx (mm)	1.43 ± 0.48	0.60 ± 0.28	0.84	0.54–1.13	< 0.001*
Ii-xMd (mm)	0.60 ± 0.20	0.42 ± 0.18	0.18	0.03–0.32	0.019*
Overbite (mm)	− 1.36 ± 0.38	− 0.51 ± 0.26	− 0.85	− 1.10 to − 0.60	< 0.001*
A’-VR (mm)	4.27 ± 0.76	0.62 ± 0.28	3.65	3.22–4.10	< 0.001*
Ls-VR (mm)	3.15 ± 1.30	1.06 ± 0.78	2.09	1.30–2.87	< 0.001*
Li-VR (mm)	− 0.24 ± 0.54	1.39 ± 0.44	− 1.63	− 2.01 to − 1.25	< 0.001*
B’-VR (mm)	− 0.72 ± 0.35	1.04 ± 0.66	− 1.78	− 2.20 to − 1.35	< 0.001*
Pg’-VR (mm)	− 0.88 ± 0.28	1.53 ± 0.34	− 2.41	− 2.65 to − 2.17	< 0.001*

*SD* standard deviation; *MD* mean difference; *CI* confidence interval

*Statistically significant at *P* < 0.05, independent-samples *t* test

**Table 6:** Intergroup comparisons of adjusted means for post-treatment cephalometric measurements with pre-treatment measurements as covariates

Variable	Control group	Treated group	Difference (treated-control)		*p* value
	Adjusted T1 Mean	Adjusted T1 Mean	Mean	95% CI	
Or-VR (mm)	51.03	53.62	2.23	1.18–3.29	< 0.001*
Ptm-VR (mm)	18.34	20.06	1.73	1.03–2.43	< 0.001*
A-VR (mm)	57.58	60.57	2.99	1.65–4.33	< 0.001*
B-VR (mm)	59.09	57.51	− 1.57	0.82–2.33	< 0.001*
Pg-VR (mm)	60.21	58.61	− 1.60	0.87–2.33	< 0.001*
A-NPerp (mm)	− 3.09	1.23	4.33	2.36–6.29	< 0.001*
Wits (mm)	− 4.01	− 0.07	3.95	1.88–6.01	< 0.001*
Co-A (mm)	74.77	77.67	2.89	1.35–4.44	< 0.001*
Co-Gn (mm)	104.78	104.29	− 0.49	− 0.19 to 1.18	0.159
SNA (°)	78.73	81.97	3.24	1.28–5.19	0.003*
SNB (°)	81.79	80.89	− 1.90	− 2.32 to 3.19	0.708
ANB (°)	3.07	− 1.08	− 4.15	1.35–6.94	0.005*
SNO (°)	55.16	57.14	1.99	0.81–3.16	0.001*
ANS-Me (mm)	55.32	55.65	0.33	− 0.08 to 0.74	0.115
SN-MP (°)	35.78	36.34	0.57	− 0.44 to 1.58	0.261
SN-PP (°)	11.39	11.59	0.19	− 0.75 to 1.14	0.681
Ar-Go-Me (°)	132.03	130.41	− 1.62	0.48–2.75	0.006*
Is-yMx (mm)	47.89	49.02	1.123	0.65–1.59	< 0.001*
Ms-yMx (mm)	18.87	19.32	0.44	0.01–0.88	0.045*
Ii-yMd (mm)	59.45	60.55	1.10	0.50–1.71	0.001*
Overjet (mm)	− 1.94	1.97	3.90	1.53–6.27	0.001*
Is-PP (°)	116.35	117.54	1.18	− 0.36 to 2.72	0.132
Ii-MP (°)	87.25	87.66	0.42	− 0.22 to 1.05	0.195
Is-xMx (mm)	25.63	25.68	0.05	− 0.24 to 0.34	0.740
Ms-xMx (mm)	19.58	20.18	0.59	− 0.03 to 1.22	0.060
Ii-xMd (mm)	36.76	36.94	0.18	− 0.02 to 0.37	0.074
Overbite (mm)	2.48	1.87	− 0.61	0.21–1.01	0.003*
A’-VR (mm)	73.69	77.37	3.67	3.13–4.21	< 0.001*
Ls-VR (mm)	79.39	81.51	2.11	1.22–3	< 0.001*
Li-VR (mm)	80.30	78.68	− 1.63	1.26–1.99	< 0.001*
B’-VR (mm)	72.03	70.25	− 1.78	1.38–2.17	< 0.001*
Pg’-VR (mm)	75.84	73.48	− 2.36	2.09–2.63	< 0.001*

*CI* confidence interval

*Statistically significant at *P* < 0.05, analysis of covariance (intention-to-treat analysis)

**Table 7:** Intergroup comparisons of adjusted means for post-treatment cephalometric measurements with pre-treatment measurements as covariates

Variable	Control group	Treated group	Difference (Treated-Control)		*p* value
	Adjusted T1 Mean	Adjusted T1 Mean	Mean	95% CI	
Or-VR (mm)	51.52	54.19	2.68	2.41–2.94	< 0.001*
Ptm-VR (mm)	18.49	20.44	1.95	1.78–2.11	< 0.001*
A-VR (mm)	57.12	60.58	3.46	3.12–3.81	< 0.001*
B-VR (mm)	58.43	56.74	− 1.69	− 2.03 to − 1.36	< 0.001*
Pg-VR (mm)	59.68	57.87	− 1.81	− 2.14 to − 1.48	< 0.001*
A-NPerp (mm)	− 3.05	1.73	4.77	4.49–5.06	< 0.001*
Wits (mm)	− 4.60	0.30	4.89	4.27–5.52	< 0.001*
Co-A (mm)	75.32	78.74	3.314	3.01–3.82	< 0.001*
Co-Gn (mm)	104.72	104.11	− 0.61	− 1.15 to − 0.07	0.028*
SNA (°)	77.87	82.06	4.19	3.68–4.69	< 0.001*
SNB (°)	81.82	80.54	− 1.28	− 1.69 to − 0.87	< 0.001*
ANB (°)	3.92	− 1.49	− 5.41	− 6.13 to − 4.69	< 0.001*
SNO (°)	55.18	57.65	2.47	2.18–2.76	< 0.001*
ANS-Me (mm)	55.52	56.02	0.51	0.19–0.83	0.003*
SN-MP (°)	35.39	35.84	0.45	− 0.21 to 1.10	0.171
SN-PP (°)	10.88	11.28	0.39	− 0.33 to 1.13	0.275
Ar-Go-Me (°)	132.25	130.07	− 2.16	− 2.68 to − 1.67	< 0.001*
Is-yMx (mm)	47.87	49.39	1.53	1.15–1.90	< 0.001*
Ms-yMx (mm)	19.11	19.64	0.53	0.24–0.82	< 0.001*
Ii-yMd (mm)	59.07	60.69	1.62	1.38–1.87	< 0.001*
Overjet (mm)	− 3.03	2.46	5.49	5.15–5.84	< 0.001*
Is-PP (°)	116.36	118.32	1.95	0.45–3.47	0.013*
Ii-MP (°)	86.95	87.57	0.62	0.23–1.01	0.003*
Is-xMx (mm)	25.57	25.70	0.12	− 0.06 to 0.31	0.173
Ms-xMx (mm)	19.19	19.98	0.78	0.49–1.08	< 0.001*
Ii-xMd (mm)	36.64	36.82	0.18	0.03–0.32	0.019*
Overbite (mm)	2.73	1.89	− 0.84	− 1.05 to − 0.63	< 0.001*
A’-VR (mm)	73.58	77.25	3.67	3.22–4.12	< 0.001*
Ls-VR (mm)	79.47	81.65	2.18	1.38–2.97	< 0.001*
Li-VR (mm)	79.97	78.35	− 1.62	− 1.98 to − 1.25	< 0.001*
B’-VR (mm)	72.06	70.30	− 1.76	− 2.14 to − 1.38	< 0.001*
Pg’-VR (mm)	75.17	72.78	− 2.39	− 2.62 to − 2.16	< 0.001*

*CI* confidence interval

*Statistically significant at *P* < 0.05, analysis of covariance (complete case analysis)

### Skeletal measurements

The maxilla showed a statistically significant advancement (A-VR) in the treated group (4.34 mm) compared with the control group (0.87 mm, *P* < 0.001). Mandibular growth was significantly restrained in the treated group (B-VR change, − 0.26 mm). The intermaxillary relationship parameters showed significant improvements in the treated group (ANB, 5.5°; Wits, 4.9 mm) compared with the control group (ANB, − 0.61°; Wits, 0.28 mm; *P* < 0.001). Vertically, the lower facial height demonstrated a significant increase in the treated group compared with the control group. The mandible showed insignificant clockwise rotation with a significant closure of the gonial angle in the treated group compared with the control group.

### Dental measurements

The maxillary and mandibular incisors demonstrated a significant protrusion in the treated group compared with the control group. Additionally, the maxillary molars showed significant mesialization and extrusion in the treated group compared with the control group. The overjet improved significantly (5.9 mm), while the overbite reduced significantly (− 1.3 mm) in the treated group compared with the control group (overjet, 0.5 mm; overbite, − 0.5; *P* < 0.001).

### Soft tissue measurements

Significant forward displacement of the upper lip and backward displacement of the mandibular soft tissue were noted in the treated group, which decreased the profile concavity.

### Harms

Mild pain was experienced on the first day following OMI placement and the first week of Alt-RAMEC.

## Discussion

This study was conducted to evaluate the skeletal, dentoalveolar, and soft tissue effects of MAMP compared with growth changes in the untreated control group, in growing patients with maxillary retrusion. The optimum time for Class III malocclusion treatment using FM is the early mixed compared with the late mixed dentition [2]. On the contrary, BAMP and MAMP can be applied successfully in the late mixed or early permanent dentition [6, 13]. Treatment at these stages enables the clinicians to keep a short post-orthopedic period of facial growth until puberty and decreases the risk of mandibular catch-up growth.

In this study, the Alt-RAMEC protocol was used to increase the maxillary protraction and decrease the treatment duration (11.9 months). These findings are not in accordance with those of Al-Mozany et al. (8.5 weeks) [10]. The difference in the results could be attributed to lower dental compensation caused by the indirect anchorage of the lower appliance to the lower teeth in their study. This would have led them to reach a positive overjet in a shorter time frame. In our study, the lower component was purely bone borne and the lower incisors advanced rather than retroclined, which made the correction more of skeletal nature and slowed down the overjet correction.

### Skeletal changes

In the treated group, the maxilla and the midface showed significant advancements which were 4 to 5 times greater than the control group. These results are consistent with the results of previous studies [10, 21–23].

A significant control over the growth of the mandible (B-VR change) was observed in the treated group (− 0.26 mm) compared with the control group (1.37 mm), suggesting that this treatment protocol restricted the growth of the mandible, as reported in previous studies [10, 13, 21, 24]. However, the effective mandibular length increased in both groups without a significant between-group difference, which corroborates with the findings of previous studies [12, 13, 21–24]. These results suggest that the increase in mandibular length is inevitable. Despite the mandibular length increasing in both groups, the use of skeletal anchorage limited the advancement of the chin point. This is also consistent with the findings of De Clerck et al. [24] who showed remodeling of the glenoid fossa and the posterior displacement of the ramus with the use of the BAMP method. There were no significant vertical changes or clockwise rotation within the treated cases in contrast to other studies using facemask with HH [25]. This is consistent with Willmann et al. [21] who also found that the HH–mentoplate combination offered superior vertical control to facemask with HH. On the contrary, a minimal counterclockwise mandibular rotation was associated with the BAMP [6, 22]. This difference could be attributed to the forces directly applied to the miniplates and not to the HH, as in the present study. The gonial angle was closed significantly in the treated group. Our findings are in agreement with the results of previous studies [6, 21, 22]. De Clerck et al. [24] explained that the decrease in the gonial angle could be attributed to the change in the shape of the mandible by the posterior displacement of the ramus without clockwise rotation of the mandible.

In the treated group, the maxillomandibular relationship was improved by the sagittal movement of the maxilla and minimal backward rotation of the mandible. In addition, the ANB angle and Wits were improved significantly. These results are in agreement with those of other studies [6, 10, 13, 22, 23].

### Dental changes

The maxillary incisors were significantly protruded in the treated group. These results are consistent with those of previous studies using hybrid appliances [10, 13, 23] and in contrast to the BAMP protocol [26, 27]. This difference could be due to the force applied to the HH, which is not a pure skeletal anchorage like the miniplates. Furthermore, the inherent flexibility of the HH arms may have allowed for a minor anchorage loss. This problem can be solved by 3-dimensional metal printing of the HH where a more rigid alloy can be used [28]. The mandibular incisors in the treated group showed a significant labial proclination compared with the control group. This change could be attributed to the increased tongue pressure on the lower incisors after eliminating the lock of the anterior crossbite. It might be also due to minimizing the lip pressure on the lower incisors by the mandibular bar [6, 21–23]. Overjet was enhanced significantly in the treated group compared with the control group. The results showed that the positive overjet was achieved by a combination of skeletal maxillary advancement and maxillary incisor protrusion. The protrusion of the lower incisors reduced the final overjet at T1 in the treated group. This result is consistent with those of previous studies [10, 12, 13, 22, 23]. The maxillary molars were significantly extruded in the treated group. Despite the molar extrusion, there was no backward rotation of the mandible because of closure in the gonial angle.

### Soft tissue changes

The skeletal and dental changes contributed to a significant profile improvement in the treated group. A significant forward movement of the upper lip was also obtained in this group, which is consistent with the results of previous studies [23, 26, 29]. The lower lip and soft tissue pogonion were restrained in the treated group in comparison with significant protrusions in the control group. These results could be attributed to the significant restriction of the mandibular growth with the negligible backward rotation of the mandible. These results agree with those of the existing literature [23, 26, 29].

## Limitations

The small sample size along with dropouts could have affected the balance between groups. However, intention-to-treat analysis was performed to deal with the missing data of the dropouts. Furthermore, the MAMP cannot be initiated before the eruption of the mandibular canine so in younger patients other approaches such as the mentoplates may need to be considered [21].

## Generalizability

Despite the study limitations, the MAMP could be an effective treatment modality for those who meet the inclusion criteria.

## Conclusions

Within the limitations of this study and high attrition rate, the results revealed that the MAMP protocol can effectively increase the maxillary forward growth with sagittal control on the mandibular forward growth. The MAMP protocol provides good vertical control, which makes it one of the treatment choices in high-angle cases. Class III concave profile was improved due to the advancement of the upper lip and restraining of the soft tissue chin of the mandible.

## Data Availability

All data generated or analyzed during this study are included in this published article.

## Abbreviations

Alt-RAMEC: Alternate rapid maxillary expansions and constrictions
BAMP: Bone-anchored maxillary protraction
C3E: Class III elastics
FM: Facemask
HH: Hybrid hyrax
MAMP: Miniscrew-anchored maxillary protraction
OMI: Orthodontic mini-implants
